# 

**Published:** 1879-12

**Authors:** 


### CONTENTS VOL. XXXIII.

Anæsthesia in Dentistry........... 35
Amaurosis following Dental Disease.................. ........... 44
Alumni Reunion..................... 44
A New Form of Tin................. 48
American Academy of Dental Science.............................. 78
An Anomalous case of Replantation................................. 92
Alumni.................................. 96
Address. By W. C. Morgan.... 141
A Case in Practice.................. 170
A Desirable Result................ 184
Alumni Association................ 226
Alveolar Abscess..................... 229
A New Method of Inserting an Artificial Crown upon a Root.. 234
Academie de Medicine et des Sciences............................... 241
American Health Primers...... 253
A Fallacious Theory Exploded.. 258
A Pleasant Method of Administering Castor Oil.................. 259
A Cause of Anæmia................ 259
A New Dental College............ 267
A New Clamp........................ 269
A New Periodical................... 270
Another Dental Law.............. 270
Address................................. 273
Anæsthesia and Anæsthetics...... 280
American Dental Association.... 210
American Society of Microscopists..................................... 312
A Well.................................. 313
A New Dental College............ 314
Anæsthesia and Anæsthetics...... 317
A Review and Criticism........ .. 343
American Health Primers........ 360
Address. By C. N. Pierce........ 361
A Stumbling Block................. 401
Aphtha of the Gums.............. 433
American Academy of Dental Science.............................. 441
Absence................................. 448
Appearance of Tongue in Disease.................................. 478
Analysis of Chlorine.............. 480
Arsenical Poisoning.............. 481
American Health Primers........ 492
Air as a Stimulant................. 523
Bibliographical...267, 359, 445, 46, 93, 496
Brain Work and Skull Growth. 351
Bibliographical...................... 532
Connecticut Valley Dental Association................................ 37
California State Dental Association................................. 95
California State Dental Association................................. 139
Commencement...................... 96
" " ........................ 140
" " ........................ 226
Correspondence....... ............... 439
Dental Nomenclature and Terminology.............................. 167
Dental Legislation................. 223
Dental Vulcanite Co.............. 264
Dental Organizations............. 369
Dental Caries......................449, 421
Disinfectants, and How to Use Them................................. 430
Dental Students...................... 514
Dr. Crawford W. Long, the Discoverer of Anæsthesia. Anæsthesia and Anæsthetics......... 518
Ether with Cod Liver Oil......... 257
Elephantine Dentals............... 262
Heredity of Color Blindness...... 42
Have We Not Too Many Dental Schools?.............................. 76
Histology of the Teeth............ 97
How to Select........................ 135
How to Cough........................ 137
Historical Sketch of the Ohio College of Dental Surgery..... 185

How We Judge Distances........ 246
Ho! For Niagara.................. 266
Hydrophobia............,............ 469
Historical Sketch of the Discovery of the Composition of Water................................ 506
How Far Can We Hear with the Telephone........................ 522
Inflammation, Abscess and Necrosis................................ 9
Illinois Dental Society......227, 138
Impacts, and the Law that Governs Them........................ 147
Inductive and Deductive Methods of Study........................ 465
India Rubber........................ 524
Josiah Bacon's Death.............. 221
Keratoscopy........................ 119
Kentucky State Dental Association.................................. 382
Meeting................................ 96
Mississippi Valley Dental Society..... ...... ................204, 183
Me and Him is Gradyates...... 375
Minutes of the Ninth Annual Meeting of the Kentucky State Dental Association...... 425
Matrimonial.............. ........... 492
Obituary...441, 448, 49, 181, 311, 358, 404
Ohio State Dental Society......... 489
Proceedings of the Tennesee Dental Society.................... 82
Prof. Ashurst on Anæsthetics.... 91
Preservation of Anatomical Specimens with Paraffine........... 136
Peterson's Dental Almanac for 1879 .................................. 219
Pathology of Tetanus.............. 215
Professional Education............ 260
Re-union of the Alumni of the Ohio College of Dental Surgery.................................. 175
Replanting Teeth.................... 223
Rip Van Winkle, Dentist......... 287
Review of the Transactions of the Maryland State Dental Society.............................. 405
Review of the Conservative Treatment of the Dental Pulp. 407
Symes on Thymol and Thymol Camphor............................ 351
Statistics of Age...... ............... 524
Scientific News........................ 525
The destruction of Dental Pulp.. 20
The Influence of the Nervous System on the Health of the Mouth.................................. 39
Tumor of the lower Jaw, the Seat of Vicarious Menstruation.................................... 128
Tetanus................................ 137
The Northern Ohio Dental Association.......................... 138
The New Departure............... 201
Transactions......................... 228
Thirty-fifth Annual Meeting of the Mississippi Dental Society. 237
The Convention of the New England Dental Societies...... 245
The Metric System................ 250
The Little Helper.................. 255
The Formation of Character... 256
The National Dental Association.................................. 266
Twentieth Meeting of the Northern Ohio Dental Association... 299
The Various Applications of Calcium Phosphate in Medicine.................................. 348
The Hygrometric Properties of Glycerine......... ................ 349
The Effects of Tobacco......... .. . 352
The Indiana Medical Protection Law.................................. 353
The Medical Profession In Rome......... ...................... 354
The Siamese Teeth............... 355
Transplanting.................. 403 356
The Results of Legislation...... 357
The Internal and External Action of Thymic Acid and Thymate of Soda................. 437
The New and the Old, and the New Departure by an Old one 493
Trophic Neuralgias................. 521
The Gardner Case—Correction.. 528
We or I, Which?..................... 48
What Is It?.......................... 5
Why do Fillings Fail?........101, 53
Where is the Fault?................ 155
Wheat Phosphates.................. 248
What Shall We Eat?.............. 252
Wisconsin......... .................... 315
Virginia State Dental Association......... ......................... 313
Very Large Salivary Calculus... 43

### *OHIO COLLEGE OF DENTAL SURGERY.*

COLLEGE STREET.

The Thirty-third Annual Session 1878-9.

### FACULTY.

JAMES TAYLOR, M. D., D. D. S.,
Principles and Practice, and Dental Hygiene.

WM. CLENDENNIN, M. D.,

Anatomy.

J. S. CASSIDY, M. D., D. D. S.,
Chemistry and Materia Medica.

J. L. CILLEY, M. D.,
Physiology and Histology.

A. O. RAWLS, D. D. S.,
Pathology and Therapeutics.

H. A. SMITH, D. D. S ,
Clinical Dentistry.

J. R. CLAYTON, D. D. S.,
Mechanical Dentistry and Microscopy.

### SPECIAL PROFESSORS.

F. H. REHWINKEL, M. D., D. D. S.,
Oral Surgery.

G. W. KEELY, D. D. S.,

Cause and Management of Irregularities of the Teeth.

C. I. KEELY, D. D. S.,
Demonstrator of Operative Dentistry.

FRANK BELL, D. D. S.,
Demonstrator of Continuous Gum Work.

S. WARDELL, D. D. S.,
Demonstrator of Carving Teeth.

ALEX. BROWN, D. D. S.,
Assistant to Chair of Chemistry.

E. G. BETTY, D. D. S., Demonstrator of Anatomy.

### CLINICAL INSTRUCTORS.

DR. S. B. BROWN. DR. F. A. HUNTER, DR. A. W. SMITH, DR. D. W. CLANCEY, DR. J. E. CRAVENS, DR. J. K. JAMISON, DR. J. I. TAYLOR, DR. F. PEABODY, DR. C. R. TAFT, DR. A. F. EMMINGER, DR. W. S. HOW.

This institution being strictly a *Dental College*, the lectures in all the branches taught, Anatomy, Physiology, Pathology, Therapeutics, and Materia Medica included, are arranged with the view to qualify the student to intelligently and successfully practice his profession.

The College Building was designed, erected, and is exclusively used for a Dental School. The lecture and clinical rooms are theretore well adapted for the uses to which they are put.

### TERMS OF GRADUATION.

A candidate for graduation must be twenty-one years of age. He must have attended two courses of lectures in a Dental or Medical College, the last of which must be in this institution. A reputable dentist in actual practice five years, inclusive of the term of pupilage, may be examined for the degree of D. D. S., after attending one full course of lectures.

### FEES.

| | |
|---|---|
| Matriculation Fee (but once) | $ 5 00 |
| Professor's Tickets for one session | 75 00 |
| Demonstrator's Ticket (for Anatomy) | 5 00 |
| Diploma Fee | 20 00 |

It is desirable that the students should matriculate and be in attendance promptly at the beginning of the term. The term begins the 1st of November, and continues sixteen weeks.

Students coming to the city should call on the Dean. Good board can be had for $3.00 to $5.00 per week.

For further information, address

***H. A. SMITH, Dean,***
286 Race St., CINCINNATI.

Aug 6t

### DIRECTORY OF DENTAL SOCIETIES.

*American Dental Association*—Meets on the first Tuesday in August, 1879, at Niagara Falls. Pres. J. H. McKellops, St. Louis, Mo.; Cor. Sec. O. A. Rawles, Lexington, Ky.; Rec. Sec. Geo. A. Cushing, Chicago, Ill.

*American Dental Convention*—Meets at Saratoga, second Tuesday in August, 1879. Pres. C. A. Marvin, Brooklyn; Rec. Sec. A. Tees, Philadelphia, Pa.

### STATE DENTAL SOCIETIES.

*California State Dental Association*—Meets first Tuesday in June, 1879. Pres. S. W. Dennis, San Francisco; Sec. S. E. Goe, San Francisco; Cor. Sec. H. E. Knox, San Francisco.

*Connecticut State Dental Society*—Pres. E. T. Payne; Rec. Sec. Geo. L. Parmelec, Hartford. Semi-annual meeting in October, 1879, at Hartford.

*Dental Society of the State of Maryland and District of Columbia*—Meets annually. Next meeting at Washington City, on the second Tuesday in October, 1879. Pres. B. F. Coy, of Baltimore; Sec. M. W. Foster, M. D.

*Georgia State Dental Society*—Meets Second Monday in July 1879, at Augusta. Pres. W. C. Wardlow, Augusta, Rec. Sec. R. A. Holliday, Atlanta, Cor. Sec. L. D. Carpenter, Atlanta.

*Illinois State Dental Society*—Meets annually. Pres. S. N. Sturgis, Quincy; Sec. E. Noyes, Chicago. Next place of meeting, Peoria, second Tuesday in May, 1879.

*Indiana State Dental Society*—Meets next year at Indianapolis, on the last Tuesday of June, 1879. Pres. T. S. Hacker, Indianapolis ; Sec. J. A. Turner, Franklin.

*Iowa State Dental Society*—Meets annually. Next meeting at Clear Lake on the second Tuesday of May, 1879. Pres. R. L. Cocheron, Burlington; Rec. Sec. E. E. Hughes, of Newton; Cor. Sec. L. C. Ingersoll, Keokuk.

*Kentucky State Dental Assseiation*—Meets annually first Tuesday in June, 1879. Next meeting in Louisville. Pres. F. Peabody, Louisville; Sec. J. S. Cassidy, Covington.

*Kansas State Dental Association*—Pres. J. D. Patterson; Sec. Wm. H. Shulze, of Atchison. Next meeting will be held at Lawrence, first Tuesday in May, 1879.

*Maine Dental Society*—Meets in August, at Thomaston. Pres. E. J. Roberts, Augusta. Sec. C. E. Hussey, Biddeford.

*Massachusetts Dental Society*—Meets semi-annually. Pres. J. H. Kidder, of Lawrence; Rec. Sec. Dwight M. Clapp, Boston; Cor. Sec. T. H. Chandler, Boston.

*Michigan State Dental Association*—Meets annually. Next meeting at Ann Arbor, March 26, 1879. Pres. Geo. L. Field, Detroit; Sec. E. C. Moore, Detroit; Treas. J. Lathrop, Detroit.

*Mississippi State Dental Association*—Will meet third Wednesday of August. 1879, at Winona. Pres. A. H. Hilzheim, Jackson; Sec. M. C. Marshall.

*Minnesota Dental Association*—Meets annually. Meets May 26. 1879. at Winona. Pres. W. F. Lewis; Sec. C. M. Bailey, Minneapolis.

*Missouri Dental Association*—Meets annually on the first Tuesday of June, 1879. Next meeting at Sweet Springs. Pres. A. N. Fuller; Sec. W. H. Eames, of St. Louis.

*Nebraska State Dental Society*—Meets annually. Next meeting July 23, 1879, at Fremont. Pres. S. H. King, Lincoln; Sec. W. F. Roseman, Fremont.

*New Jersey State Dental Society*—Meets annually. Next meeting at Long Branch, on the second Tuesday in July, 1879. Pres. T. B. Welch, Newark; Sec. C. A. Meeker, Newark.

*New Hampshire Dental Society*—Pres. D. W. Eagerly, Farmington; Sec. C. W. Clerment, Manchester.

*North Carolina State Dental Society*—Meets annually. Next meeting at Raleigh, first Wednesday in Sept.., 1879. Pres. V. E. Turner; Sec. D. A. Robinson.

*Ohio State Dental Society.*—Meets annually at Columbus, first Wednesday of December, 1879. Pres. D. R. Jennings, Cleveland; Rec. Sec. W. H. Sillito, Xenia; Cor. Sec., A. L. Brown, Chillicothe.

*Oregon State Dental Society*—Meets annually. Pres. J. R. Cardwell, Portland; Rec. Sec. S. J. Barber.

*Pennsylvania State Dental Society*—Meets in Bedford next, Tuesday, July 30, 1879. Pres. J. S. King; Rec. Sec. S. H. Guilford; Cor. Sec. M. H. Webb.

*Rhode Island Dental Association*—Pres. A. W. Buckland, Woonsocket; Sec. L. L. Buckland, Providence.

*South Carolina State Dental Association*—Meets annually. Pres. J. B. Patrick, Charleston; Sec. G. F. S. Wright, Columbia; Cor. Sec. B. H. Teagne, Aikin. Next meeting at Charleston, the second Tuesday of July, 1879.

*Tennesee Dental Association*—Meets semi-annually. Next meeting at Nashville, fourth Wednesday in June, 1879. Pres. L. G. Noel; Rec. Sec. H. E. Beach; Cor. Sec. J. C. Ross, Nashville.

*Vermont State Dental Society*—Meets annually. Pres. J. L. Perkins, St. Johnsburg; Sec. C. D. Newell, St. Johnsburg.

*Virginia State Dental Association*—Meets in Richmond, December 14. Pres. J. H Moore; Sec. L. M. Cowardin, Richmond.

*Wisconsin State Dental Association*—Meets annually. Next meeting at Milwaukee, in July 8, 1879. Pres. J. M. Baker, of Elkhorn; Sec. M. T. Moore, La Crosse.

*The Dental Society of the State of New York.*—Meets annually on the second Wednesday in May. Next session at Albany, May 14, 1879. Pres. A. M. Holmes, Morrisville; Rec. Sec. S. A. Freeman, Buffalo; Cor. Sec. W. H. Atkinson, New York.

### LOCAL SOCIETIES.

*American Academy of Dental Science.*—Meets Annually in Boston, Mass. Next Meeting on Wednesday, Oct. 59, 1879. Pres. E. G. Tucker; Rec. Sec. E. P. Bradbury; Cor. Sec. E. N. Harris, Boston.

*Connecticut Valley Dental Association.*—Meets in June 1879, in Boston. Pres. C. A Bracket, Newport, R. I.; Sec, C. T. Stockwell, Springfield, Mass.

*East Tennessee Dental Association*—Meets annually. Next meeting will be held at Morristown, Tenn., on the First Wednesday of Aug., 1879. Pres. Thos. I. Speck; Sec. J. B. Williams, Maryville.

*Mississippi Valley Dental Association*—Meets annually at Cincinnati, on first Wednesday in March, 1880. Pres. F. A. Hunter, Cincinnati; Sec. Wm. Taft, Cincinnati.

*Nashville Dental Association*—Meets on the second Tuesday of each month. Pres. R. R. Freeman; Sec. L. G. Noel, Nashville.

*Northern Ohio Dental Association*—Meets annually. Next meeting at Cleveland, on the second Tuesday in May, 1879. Pres. J. W. Lyder, Akron, O; Sec. L. C. Kelsey Elyria.

*Ohio College Dental Association*—Meets annually, Tuesday, March 4, 1879, at Cincinnati. Pres. F. A. Hunter, Cincinnati; Rec. Sec. H. A Smith, Cincinnati

*Odontographic Society of Pennsylvania*—Meets on the first Wednesday of each month, at 8 o'clock p. m., in the Philadelphia Dental College. Pres. M. Lukins; Rec. Sec. E. L. Hewitt.

*Odontological Society of New York City*—Meets the second Tuesday of each month. Pres. A. L. Northrup; Rec. Sec. Wm. Jarvie, Jr.

*Pittsburgh Dental Society*—Meets the second Tuesday evening of each month. Pres. N. W. Aithen; Sec. L. Depriz.

*Southern Dental Association*—Meets annually. Next meeting at Augusta, Ga., July 8, 1879. Pres. F. J. S. Gorgas, Baltimore; Rec. Sec. E. L. Chisholm, Tuscaloosa, Ala.

### EXCHANGES.

ST. LOUIS MEDICAL AND SURGICAL JOURNAL.
THE PACIFIC MEDICAL AND SURICAL JOURNAL, SAN FRANCISCO.
CINCINNATI MEDICAL ADVANCE.
CINCINNATI LANCET AND CLINIC.
DENTAL COSMOS, PHILADELPHIA.
LADIES' REPOSITORY, CINCINNATI.
CHICAGO MEDICAL EXAMINER.
THE DETROIT REVIEW OF MEDICINE AND PHARMACY.
MEDICAL RECORD, N. Y.
NASHVILLE JOURNAL OF MEDICINE AND SURGERY.
AMERICAN JOURNAL OF DENTAL SCIENCE, BALTIMORE.
THE UNITED STATES MEDICAL AND SURGICAL JOURNAL, CHICAGO.
BOSTON JOURNAL OF CHEMISTRY.
CANADA JOURNAL OF DENTAL SCIENCE, MONTREAL.
INDIANA JOURNAL OF MEDICINE.
MEDICAL AND SURGICAL REPORTER, SALEM, OREGON.
MISSOURI DENTAL JOURNAL, ST. LOUIS.
ECLECTIC MEDICAL JOURNAL, CINCINNATI.
THE SANITARAN, NEW YORK CITY.
BRITISH JOURNAL OF DENTAL SCIENCE, LONDON.
THE MONTHLY REVIEW OF DENTAL SURGERY, LONDON.
L'ART DENTOIRE, PARIS.
LE PROGRESS DENTOIRE, PARIS.
DEUTISCHE VERTELJAHRSSCHRIFT, LEIPZIG.

### THE DENTAL COLLEGE
### OF THE
### UNIVERSITY OF MICHIGAN.

The Fifth Annual Session of this Institution will commence on the 1st of October and close on the last Wednesday of March, thus making a course of six months. The regular course of instruction commences at once, and will proceed through the term, with the customary vacation.

The students in this department will receive instructions in Anatomy, Physiology, Pathology, Chemistry, Materia Medica, Therapeutics and Surgery, from the Professors of their respective branches in the *Department of Medicine and Surgery* of the University, when lectures commence, and continue the same as with the Dental College. There will also, in addition, be a special course upon each of these branches. These courses will each embrace from fifteen to twenty lectures.

---

### *FACULTY OF THE DENTAL DEPARTMENT.*

J. B. ANGELL, LL. D., - - - - - - - - - President
J. TAFT, D. D. S., - Principles and Practice of Operative Dentistry
CORYDON L. FORD, - - - - - Anatomy and Physiology
J. A. WATLING, D. D. S., - - Clinical and Mechanical Dentistry
W. H. DORRANCE, D. D. S., Demonstrator of Mechanical Dentistry

### *SPECIAL COURSES.*

A. B. PALMER, M. D., - - - - - - Dental Pathology
DONALD MACLEAN, M. D., - - - - - - Oral Surgery
E. S. DUNSTER, M. D.,
Diseases of Women and Children with reference to the Teeth
GEO. E. FROTHINGHAM, M. D. - - - - Dental Therapeutics

*Geo. L. Field, D. D. S., will give Special Instruction in Continuous Gum Work.*

Students should be promptly present at the College on Tuesday, September 30th, at ten o'clock, A. M., to make the preliminary arrangements for entering upon regular work on the morning of October 1st. Seats in the lecture room are assigned by selection to students in the order of registration on the Steward's books; and each student is expected to occupy during the session such seat as he may select. Students on arriving in Ann Arbor, should call at the Steward's office.

### *CONDITIONS OF GRADUATION.*

The candidate must be twenty-one years of age. He must furnish evidence of good moral character.

He must devote three years to the study of his profession. He must attend two full courses of lectures in the Dental College, or one course elsewhere and the last one here, and we recommend that he attend three courses regularly.

He must sustain an examination satisfactory to the Faculty in all the branches taught.

A graduate of the Medical College may enter the senior class, and if found qualified, may graduate after one year has been devoted to the study of dentistry.

### FEES AND EXPENSES.

The fees, which must be paid in advance, are as follows:

RESIDENTS OF MICHIGAN.—Matriculation fee, $10.00; annual dues, $20.00.

NON-RESIDENTS.—Matriculation, $25.00; annual dues, $25.00.

GRADUATION FEE.—For all alike, $10.00. The admission fee is paid but once, and entitles the student the privileges of permanent membership in any department of the University. The annual tax is paid the first year, aud every year thereafter while at the University.

For further particulars, address the Dean of the Dental College, Ann Arbor, Michigan.

J. TAFT, Dean.

May-6t

### OHIO COLLEGE of DENTAL SURGERY

### 29 College Street.

### Thirty-Fourth Annual Session 1879-80

---

### FACULTY.

JAS. TAYLOR, M. D., D. D. S., - Principles and Practice and Dental Hygiene.
WM. CLENDENIN, M. D., - - - - - - Descriptive Anatomy.
J. S. CASSIDY, M. D., D. D. S., - - - Chemistry and Materia Medica.
J. L. CILLEY, M. D., - - - - - - Physiology and Histology.
H. A. SMITH, D. D. S., - - - - - - - - Clinical Dentistry.
A. O. RAWLS, D. D. S., - - - - - - Pathology and Therapeutics.
J. R. CLAYTON, D. D. S., - - Mechanical Dentistry and Metallurgy.
WM. TAFT, M. D., D. D. S., - - Oral Surgery and Practical Anatomy.

### DEMONSTRATORS.

——————————, - - - - - Demonstrator of Operative Dentistry
H. L. MOORE, D. D. S., - Assistant Demonstrator of Operative Dentistry.
E. W. ANDERSON, D. D. S., - - - Demonstrator of Mechanical Dentistry.
C. W. WARDLE, D. D. S., - - - - Instructor in Continuous Gum Work.
OSCAR HEISE, D. D. S., - - - - - Assistant to Chair of Chemistry.

### CLINICAL INSTRUCTORS.

Dr. F. H. REHWINKEL,

Dr. G. W. KEELY,

Dr. J. I. TAYLOR,

Dr. F. PEABODY,

Dr. E. OSMOND,

Dr. N. S. HOFF,

Dr. A. W. SMITH,

Dr. F. A. HUNTER,

Dr. S. WARDELL,

Dr. C. R. TAFT,

Dr. D. W. CLANCEY.

Dr. W. S. HOW.

This institution being *strictly a Dental College*, the lectures in all the branches taught, Anatomy, Physiology, Pathology, Therapeutics and Materia Medica included, are arranged with the view to qualify the student to intelligently practice his profession.

The College Building was designed, erected and is *exclusively used* for a Dental School. The location is central and within a short distance of the Post Office, Music and Exposition Hall, Hospital and other public structures. Located in one of the most populous cities, where there is no other Dental College, the supply of patients for clinics is abundant. Superior advantages are thus afforded students for practical work.

### REQUIREMENTS FOR GRADUATION.

A candidate for graduation must be twenty-one years of age. He must have attended two courses of lectures in a Dental or Medical College, the last of which must be in this institution. A reputable dentist in actual practice five years, inclusive of the term of pupilage, may be examined for the degree of D. D. S., after attending one full course of lectures.

### FEES.

| | |
|---|---|
| Matriculation Fee (but once) - - - - - - - | $ 5 00 |
| Professor's Tickets for one session, - - - - - | 75 00 |
| Demonstrator's Ticket (for Anatomy) - - - - - - | 5 00 |
| Diploma Fee, - - - - - - - - - - | 20 00 |

The session begins the 22d of October, and continues until the first of March. The regular lectures begins at once, and students should be prompt in matriculating.

Good Board can be had for $3 50 to $6 00 per week.

For further information, address

***H. A. SMITH, Dean,***

286 Race Street, Cincinnati.

### THE DENTAL COLLEGE
### OF THE
### UNIVERSITY OF MICHIGAN.

The Fifth Annual Session of this Institution will commence on the 1st of October and close on the last Wednesday of March, thus making a course of six months. The regular course of instruction commences at once, and will proceed through the term, with the customary vacation.

The students in this department will receive instructions in Anatomy, Physiology, Pathology, Chemistry, Materia Medica, Therapeutics and Surgery, from the Professors of their respective branches in the *Department of Medicine and Surgery* of the University, when lectures commence, and continue the same as with the Dental College. There will also, in addition, be a special course upon each of these branches. These courses will each embrace from fifteen to twenty lectures.

---

### *FACULTY OF THE DENTAL DEPARTMENT.*

J. B. ANGELL, LL. D., - - - - - - - - President
J. TAFT, D. D. S., - Principles and Practice of Operative Dentistry
CORYDON L. FORD, - - - - Anatomy and Physiology
J. A. WATLING, D. D. S., - - Clinical and Mechanical Dentistry
W. H. DORRANCE, D. D. S., Demonstrator of Mechanical Dentistry

### *SPECIAL COURSES.*

A. B. Palmer, M. D., - - - - - - - Dental Pathology
Donald Maclean, M. D., - - - - - - Oral Surgery
E. S. Dunster, M. D.,
Diseases of Women and Children with reference to the Teeth
Geo. E. Frothingham, M. D. - - - - Dental Therapeutics
*Geo. L. Field, D. D. S., will give Special Instruction in Continuous Gum Work.*

Students should be promptly present at the College on Tuesday, September 30th, at ten o'clock, A. M., to make the preliminary arrangements for entering upon regular work on the morning of October 1st. Seats in the lecture room are assigned by selection to students in the order of registration on the Steward's books; and each student is expected to occupy during the session such seat as he may select. Students on arriving in Ann Arbor, should call at the Steward's office.

### *CONDITIONS OF GRADUATION.*

The candidate must be twenty-one years of age. He must furnish evidence of good moral character.

He must devote three years to the study of his profession. He must attend two full courses of lectures in the Dental College, or one course elsewhere and the last one here, and we recommend that he attend three courses regularly.

He must sustain an examination satisfactory to the Faculty in all the branches taught.

A graduate of the Medical College may enter the senior class, and if found qualified, may graduate after one year has been devoted to the study of dentistry.

### FEES AND EXPENSES.

The fees, which must be paid in advance, are as follows:

Residents of Michigan.—Matriculation fee, $10 00; annual dues, $20.00.

Non-Residents.—Matriculation, $25.00; annual dues, $25.00.

Graduation Fee.—For all alike, $10.00. The admission fee is paid but once, and entitles the student the privileges of permanent membership in any department of the University. The annual tax is paid the first year, aud every year thereafter while at the University.

For further particulars, address the Dean of the Dental College, Ann Arbor, Michigan.

J. TAFT, Dean.

May-6t

*New Jersey State Dental Society*—Meets annually. Next meeting at Long Branch. on Wednesday, July, 16th, 1879. Pres. Chas. A. Meeker, Newark; Sec. C. S. Stockton, Newark.

*New Hampshire Dental Society*—Pres. D. W. Eagerly, Farmington; Sec. C. W. Clerment, Manchester.

*North Carolina State Dental Society*—Meets annually. Next meeting at Raleigh, first Wednesday in Sept.., 1879. Pres. V. E. Turner; Sec. D. A. Robinson.

*Northern Ohio Dental Association.*—Meets annually. Next mee'ing at Akron, second Tuesday and Wednesday of May, 1880. Pres. J. W. Lyder, Akron; Sec. L. C, Kelsey, Elyria.

*Ohio State Dental Society.*—Meets annually at Columbus, first Wednesday of December, 1879. Pres. D. R. Jennings, Cleveland; Rec. Sec. W. H. Sillito, Xenia; Cor. Sec., A. L. Brown, Chillicothe.

*Oregon State Dental Society*—Meets annually. Pres. J. R. Cardwell, Portland; Rec. Sec. S . J. Barber.

*Pennsylvania State Dental Society*—Meets at Delaware Water Gap, next, Tuesday, July 30, 1879. Pres. C. N. Pierce; Rec. Sec. S. H. Guilford; Cor. Sec. G. W. Klump.

*Rhode Island Dental Association*—Pres. A. W. Buckland, Woonsocket; Sec. L. L. Buckland, Providence.

*South Carolina State Dental Association*—Mee annually. Pres. J. B. Patrick, Charleston; Sec. G. F. S. Wright, Columbia; Cor. Sec. B. H. Teagne, Aikin. Next meeting at Charleston, the second Tuesday of July, 1879.

*Tennesee Dental Association*—Meets semi-annually. Next meeting at Nashville, fourth Wednesday in June, 1879. Pres. L. G. Noel; Rec. Sec. H. E. Beach; Cor. Sec. J. C. Ross, Nashville.

*Vermont State Dental Society*—Meets annually. Pres. J. L. Perkins, St. Johnsburg; Sec. C. D. Newell, St. Johnsburg.

*Virginia State Dental Association*—Meets in Charlottsville, Tuesday, August 19, 1879. Pres. J. Hal Moore; Sec. L. M. Cowardin, Richmond.

*Wisconsin State Dental Association*—Meets annually. Next meeting at Milwaukee, in July 8, 1879. Pres. J. M. Baker, of Elkhorn; Sec. M. T. Moore, La Crosse.

*The Dental Society of the State of New York.*—Mee s annually on the second Wednesday in May. Next session at Albany, May 14, 1879. Pres. A. M. Holmes, Morrisville; Rec. Sec. S. A. Freeman, Buffalo; Cor. Sec. W. H. Atkinson, New York.

### LOCAL SOCIETIES.

*American Academy of Dental Science.*—Meets Annually in Boston, Mass. Next Meeting on Wednesday, Oct. 59, 1879. Pres. E. G. Tucker; Rec. Sec. E. P. Bradbury; Cor. Sec. E. N. Harris, Boston.

*Connecticut Valley Dental Association.*—Meets in June 1879, in Boston. Pres. C. A Bracket, Newport, R. I.; Sec, C. T. Stockwell, Springfield, Mass.

*East Tennessee Dental Association*—Meets annually. Next meeting will be held a Morristown, Tenn., on the First Wednesday of Aug , 1879. Pres. Thos. I. Speck Sec. J. B. Williams, Maryville.

*Mississippi Valley Dental Association*—Meets annually at Cincinnati, on first Wednesday in March, 1880. Pres. F. A. Hunter, Cincinnati; Sec. Wm. Taft, Cincinnati.

*Nashville Dental Association*—Meets on the second Tuesday of each month. Pres. R. R. Freeman; Sec. L. G. Noel, Nashville.

*Northern Ohio Dental Association*—Meets annually. Next meeting at Cleveland, on the second Tuesday in May, 1879. Pres. J. W. Lyder, Akron, O; Sec. L. C. Kel sey Elyria.

*Ohio College Dental Association*—Meets annually, Tuesday, March 4, 1879, at Cincinnati. Pres. F. A. Hunter, Cincinnati; Rec. Sec. H. A. Smith. Cincinnati

*Odontographic Society of Pennsylvania*—Meets on the first Wednesday of each month at 8 o'clock p. m., in the Philadelphia Dental College. Pres. M. Lukins; Rec Sec. E. L. Hewitt.

*Odontological Society of New York City*—Meets the second Tuesday of each month Pres. A. L. Northrup; Rec. Sec. Wm. Jarvie, Jr.

*Pittsburgh Dental Society*—Meets the second Tuesday evening of each month. Pres. N. W. Aithen; Sec. L. Depriz.

*Southern, Dental Association*—Meets annually. Next meeting at Augusta, Ga., July 8, 1879, Pres. F. J. S. Gorgas, Baltimore; Rec. Sec. E. L. Chisholm, Tuscaloosa, Ala.

### EXCHANGES.

ST. LOUIS MEDICAL AND SURGICAL JOURNAL.
THE PACIFIC MEDICAL AND SURICAL JOURNAL, SAN FRANCISCO.
CINCINNATI MEDICAL ADVANCE.
CINCINNATI LANCET AND CLINIC.
DENTAL COSMOS, PHILADELPHIA.
LADIES' REPOSITORY, CINCINNATI.
CHICAGO MEDICAL EXAMINER.
THE DETROIT REVIEW OF MEDICINE AND PHARMACY.
MEDICAL RECORD, N. Y.
NASHVILLE JOURNAL OF MEDICINE AND SURGERY.
AMERICAN JOURNAL OF DENTAL SCIENCE, BALTIMORE.
THE UNITED STATES MEDICAL AND SURGICAL JOURNAL, CHICAGO
BOSTON JOURNAL OF CHEMISTRY.
CANADA JOURNAL OF DENTAL SCIENCE, MONTREAL.
INDIANA JOURNAL OF MEDICINE.
MEDICAL AND SURGICAL REPORTER, SALEM, OREGON
MISSOURI DENTAL JOURNAL, ST. LOUIS.
E LECTIC MEDICAL JOURNAL, CINCINNATI.
THE SANITARAN, NEW YORK CITY.
BRITISH JOURNAL OF DENTAL SCIENCE, LONDON.
THE MONTHLY REVIEW OF DENTAL SURGERY, LONDON.
L'ART DENTOIRE, PARIS.
LE PROGRESS DENTOIRE, PARIS.
DEUTISCHE VERTELJAHRSSCHRIFT, LEIPZIG.

### OHIO COLLEGE of DENTAL SURGERY

### 29 College Street.

### Thirty-Fourth Annual Session 1879-80

---

### FACULTY.

JAS. TAYLOR, M. D., D. D. S., - Principles and Practice and Dental Hygiene.
WM. CLENDENIN, M. D., - - - - - - Descriptive Anatomy.
J. S. CASSIDY, M. D., D. D. S., - - - Chemistry and Materia Medica.
J. L. CILLEY, M. D., - - - - - - - Physiology and Histology.
H. A. SMITH, D. D. S., - - - - - - - - Clinical Dentistry.
A. O. RAWLS, D. D. S., - - - - - - Pathology and Therapeutics.
J. R. CLAYTON, D. D. S., - - Mechanical Dentistry and Metallurgy.
WM. TAFT, M. D., D. D. S, - - Oral Surgery and Practical Anatomy.

### DEMONSTRATORS.

————————, - - - - - Demonstrator of Operative Dentistry.
H. L. MOORE, D. D. S., - Assistant Demonstrator of Operative Dentistry.
E. W. ANDERSON, D. D. S., - - Demonstrator of Mechanical Dentistry.
C. W. WARDLE, D. D. S., - - - Instructor in Continuous Gum Work.
OSCAR HEISE, D. D. S., - - - - Assistant to Chair of Chemistry.

### CLINICAL INSTRUCTORS.

DR. F. H. REHWINKEL,

DR. G. W. KEELY,

DR. J. L. TAYLOR,

DR. F. PEABODY,

DR. E. OSMOND,

DR. N. S. HOFF,

DR. A. W. SMITH,

DR. F. A. HUNTER,

DR. S. WARDELL,

DR. C. R. TAFT,

DR. D. W. CLANCEY.

DR. W. S. HOW.

This institution being *strictly a Dental College*, the lectures in all the branches taught, Anatomy, Physiology, Pathology, Therapeutics and Materia Medica included, are arranged with the view to qualify the student to intelligently practice his profession.

The College Building was designed, erected and is *exclusively used* for a Dental School. The location is central and within a short distance of the Post Office, Music and Exposition Hall, Hospital and other public structures. Located in one of the most populous cities, where there is no other Dental College, the supply of patients for clinics is abundant. Superior advantages are thus afforded students for practical work.

### REQUIREMENTS FOR GRADUATION.

A candidate for graduation must be twenty-one years of age. He must have attended two courses of lectures in a Dental or Medical College, the last of which must be in this institution. A reputable dentist in actual practice five years, inclusive of the term of pupilage, may be examined for the degree of D. D. S., after attending one full course of lectures.

### FEES.

| | |
|---|---|
| Matriculation Fee (but once) | $ 5 00 |
| Professor's Tickets for one session, | 75 00 |
| Demonstrator's Ticket (for Anatomy) | 5 00 |
| Diploma Fee, | 20 00 |

The session begins the 22d of October, and continues until the first of March. The regular lectures begins at once, and students should be prompt in matriculating.

Good Board can be had for $3 50 to $6 00 per week.

For further information, address

***H. A. SMITH, Dean,***
286 Race Street, Cincinnati.

### THE DENTAL COLLEGE
### OF THE
### UNIVERSITY OF MICHIGAN.

The Fifth Annual Session of this Institution will commence on the 1st of October and close on the last Wednesday of March, thus making a course of six months. The regular course of instruction commences at once, and will proceed through the term, with the customary vacation.

The students in this department will receive instructions in Anatomy, Physiology, Pathology, Chemistry, Materia Medica, Therapeutics and Surgery, from the Professors of their respective branches in the *Department of Medicine and Surgery* of the University, when lectures commence, and continue the same as with the Dental College. There will also, in addition, be a special course upon each of these branches. These courses will each embrace from fifteen to twenty lectures.

---

### *FACULTY OF THE DENTAL DEPARTMENT.*

J. B. ANGELL, LL. D., - - - - - - - - President
J. TAFT, D. D. S., - Principles and Practice of Operative Dentistry
CORYDON L. FORD, M. D., D, D. S. - Anatomy and Physiology
J. A. WATLING, D. D. S., - - Clinical and Mechanical Dentistry
W. H. DORRANCE, D. D. S., Demonstrator of Mechanical Dentistry

### *SPECIAL COURSES.*

A. B. Palmer, M. D., - - - - - - - Dental Pathology
Donald Maclean, M. D., - - - - - - - Oral Surgery
E. S. Dunster, M. D.,
Diseases of Women and Children with reference to the Teeth
Geo. E. Frothingham, M. D. - - - - Dental Therapeutics
*Geo. L. Field, D. D. S., will give Special Instruction in Continuous Gum Work.*

Students should be promptly present at the College on Tuesday, September 30th, at ten o'clock, A. M., to make the preliminary arrangements for entering upon regular work on the morning of October 1st. Seats in the lecture room are assigned by selection to students in the order of registration on the Steward's books; and each student is expected to occupy during the session such seat as he may select. Students on arriving in Ann Arbor, should call at the Steward's office.

### *CONDITIONS OF GRADUATION.*

The candidate must be twenty-one years of age. He must furnish evidence of good moral character.

He must devote three years to the study of his profession. He must attend two full courses of lectures in the Dental College, or one course elsewhere and the last one here, and we recommend that he attend three courses regularly.

He must sustain an examination satisfactory to the Faculty in all the branches taught.

A graduate of the Medical College may enter the senior class, and if found qualified, may graduate after one year has been devoted to the study of dentistry.

### **FEES AND EXPENSES.**

The fees, which must be paid in advance, are as follows:

Residents of Michigan.—Matriculation fee, $10.00; annual dues, $20.00.

Non-Residents.—Matriculation, $25.00; annual dues, $25.00.

Graduation Fee.—For all alike, $10.00. The admission fee is paid but once, and entitles the student the privileges of permanent membership in any department of the University. The annual tax is paid the first year, aud every year thereafter while at the University.

For further particulars, address the Dean of the Dental College, Ann Arbor, Michigan. J. TAFT, Dean.

May-6t

### OHIO COLLEGE of DENTAL SURGERY

### 29 College Street.

### Thirty-Fourth Annual Session 1879-80

---

### FACULTY.

| | |
|---|---|
| AJS. TAYLOR, M. D., D. D. S., | Principles and Practice and Dental Hygiene. |
| WM. CLENDENIN, M. D., | Descriptive Anatomy. |
| J. S. CASSIDY, M. D., D. D. S., | Chemistry and Materia Medica. |
| J. L. CILLEY, M. D., | Physiology and Histology. |
| H. A. SMITH, D. D. S., | Clinical Dentistry. |
| A. O. RAWLS, D. D. S., | Pathology and Therapeutics. |
| J. R. CLAYTON, D. D. S., | Mechanical Dentistry and Metallurgy. |
| WM. TAFT, M. D., D. D. S., | Oral Surgery and Practical Anatomy. |
| OTTO ARNOLD, D. D. S., | Demonstrator of the use of Nitrous Oxide Gas. |

### DEMONSTRATORS.

| | |
|---|---|
| C. I. KEELY, | Demonstrator of Operative Dentistry. |
| H. L. MOORE, D. D. S., | Assistant Demonstrator of Operative Dentistry. |
| E. W. ANDERSON, D. D. S., | Demonstrator of Mechanical Dentistry. |
| C. W. WARDLE, D. D. S., | Instructor in Continuous Gum Work. |
| OSCAR HEISE, D. D. S., | Assistant to Chair of Chemistry. |

### CLINICAL INSTRUCTORS.

Dr. F. H. REHWINKEL,

Dr. G. W. KEELY,

Dr. J. I. TAYLOR,

Dr. F. PEABODY,

Dr. E. OSMOND,

Dr. N. S. HOFF,

Dr. J. E. CRAVENS,

Dr. A. W. SMITH,

Dr. F. A. HUNTER,

Dr. S. WARDELL,

Dr. C. R. TAFT,

Dr. D. W. CLANCEY.

Dr. W. S. HOW.

Dr. T. S HACKER.

This institution being *strictly a Dental College*, the lectures in all the branches taught, Anatomy, Physiology, Pathology, Therapeutics and Materia Medica included, are arranged with the view to qualify the student to intelligently pactrice his profession.

The College Building was designed, erected and is *exclusively used* for a Dental School. The location is central and within a short distance of the Post Office, Music and Exposition Hall, Hospital and other public structures. Located in one of the most populous cities, where there is no other Dental College, the supply of patients for clinics is abundant. Superior advantages are thus afforded students for practical work.

### REQUIREMENTS FOR GRADUATION.

A candidate for graduation must be twenty-one years of age. He must have attended two courses of lectures in a Dental or Medical College, the last of which must be in this institution. A reputable dentist in actual practice five years, inclusive of the term of pupilage, may be examined for the degree of D. D. S., after attending one full course of lectures.

### FEES.

| | |
|---|---|
| Matriculation Fee (but once) | $ 5 00 |
| Professor's Tickets for one session, | 75 00 |
| Demonstrator's Ticket (for Anatomy) | 5 00 |
| Diploma Fee, | 20 00 |

The session begins the 22d of October, and continues until the first of March. The regular lectures begins at once, and students should be prompt in matriculating.

Good Board can be had for $3 50 to $6 00 per week.

For further information, address

***H. A. SMITH, Dean,***

286 Race Street, Cincinnati.

### THE DENTAL COLLEGE
### OF THE
### UNIVERSITY OF MICHIGAN.

The Fifth Annual Session of this Institution will commence on the 1st of October and close on the last Wednesday of March, thus making a course of six months. The regular course of instruction commences at once, and will proceed through the term, with the customary vacation.

The students in this department will receive instructions in Anatomy, Physiology, Pathology, Chemistry, Materia Medica, Therapeutics and Surgery, from the Professors of their respective branches in the *Department of Medicine and Surgery* of the University, when lectures commence, and continue the same as with the Dental College. There will also, in addition, be a special course upon each of these branches. These courses will each embrace from fifteen to twenty lectures.

---

### *FACULTY OF THE DENTAL DEPARTMENT.*

J. B. ANGELL, LL. D., - - - - - - - - - President

J. TAFT, D. D. S., - Principles and Practice of Operative Dentistry

CORYDON L. FORD, M. D., D, D. S. - Anatomy and Physiology

J. A. WATLING, D. D. S., - - Clinical and Mechanical Dentistry

W. H. DORRANCE, D. D. S., Demonstrator of Mechanical Dentistry

### *SPECIAL COURSES.*

A. B. PALMER, M. D., - - - - - - - Dental Pathology

DONALD MACLEAN, M. D., - - - - - - Oral Surgery

E. S. DUNSTER, M. D.,
Diseases of Women and Children with reference to the Teeth

GEO. E. FROTHINGHAM, M. D. - - - - Dental Therapeutics

*Geo. L. Field, D. D. S., will give Special Instruction in Continuous Gum Work.*

Students should be promptly present at the College on Tuesday, September 30th, at ten o'clock, A. M., to make the preliminary arrangements for entering upon regular work on the morning of October 1st. Seats in the lecture room are assigned by selection to students in the order of registration on the Steward's books; and each student is expected to occupy during the session such seat as he may select. Students on arriving in Ann Arbor, should call at the Steward's office.

### *CONDITIONS OF GRADUATION.*

The candidate must be twenty-one years of age. He must furnish evidence of good moral character.

He must devote three years to the study of his profession. He must attend two full courses of lectures in the Dental College, or one course elsewhere and the last one here, and we recommend that he attend three courses regularly.

He must sustain an examination satisfactory to the Faculty in all the branches taught.

A graduate of the Medical College may enter the senior class, and if found qualified, may graduate after one year has been devoted to the study of dentistry.

### FEES AND EXPENSES.

The fees, which must be paid in advance, are as follows:

RESIDENTS OF MICHIGAN.—Matriculation fee, $10.00; annual dues, $20.00.

NON-RESIDENTS.—Matriculation, $25.00; annual dues, $25.00.

GRADUATION FEE.—For all alike, $10.00. The admission fee is paid but once, and entitles the student the privileges of permanent membership in any department of the University. The annual tax is paid the first year, aud every year thereafter while at the University.

For further particulars, address the Dean of the Dental College, Ann Arbor, Michigan.

J. TAFT, Dean.

May-6t

### THE DENTAL COLLEGE
### OF THE
### UNIVERSITY OF MICHIGAN.

The Fifth Annual Session of this Institution will commence on the 1st of October and close on the last Wednesday of March, thus making a course of six months. The regular course of instruction commences at once, and will proceed through the term, with the customary vacation.

The students in this department will receive instructions in Anatomy, Physiology, Pathology, Chemistry, Materia Medica, Therapeutics and Surgery, from the Professors of their respective branches in the *Department of Medicine and Surgery* of the University, when lectures commence, and continue the same as with the Dental College. There will also, in addition, be a special course upon each of these branches. These courses will each embrace from fifteen to twenty lectures.

---

### *FACULTY OF THE DENTAL DEPARTMENT.*

J. B. ANGELL, LL. D., - - - - - - - - President
J. TAFT, D. D. S., - Principles and Practice of Operative Dentistry
CORYDON L. FORD, M. D., D, D. S. - Anatomy and Physiology
J. A. WATLING, D. D. S., - - Clinical and Mechanical Dentistry
W. H. DORRANCE, D. D. S., Demonstrator of Mechanical Dentistry

### *SPECIAL COURSES.*

A. B. PALMER, M. D., - - - - - - Dental Pathology
DONALD MACLEAN, M. D., - - - - - - Oral Surgery
E. S. DUNSTER, M. D.,
Diseases of Women and Children with reference to the Teeth
GEO. E. FROTHINGHAM, M. D. - - - - Dental Therapeutics

*Geo. L. Field, D. D. S., will give Special Instruction in Continuous Gum Work.*

Students should be promptly present at the College on Tuesday, September 30th, at ten o'clock, A. M., to make the preliminary arrangements for entering upon regular work on the morning of October 1st. Seats in the lecture room are assigned by selection to students in the order of registration on the Steward's books; and each student is expected to occupy during the session such seat as he may select. Students on arriving in Ann Arbor, should call at the Steward's office.

### *CONDITIONS OF GRADUATION.*

The candidate must be twenty-one years of age. He must furnish evidence of good moral character.

He must devote three years to the study of his profession. He must attend two full courses of lectures in the Dental College, or one course elsewhere and the last one here, and we recommend that he attend three courses regularly.

He must sustain an examination satisfactory to the Faculty in all the branches taught.

A graduate of the Medical College may enter the senior class, and if found qualified, may graduate after one year has been devoted to the study of dentistry.

### FEES AND EXPENSES.

The fees, which must be paid in advance, are as follows:

RESIDENTS OF MICHIGAN.—Matriculation fee, $10.00; annual dues, $20.00.

NON-RESIDENTS.—Matriculation, $25.00; annual dues, $25.00.

GRADUATION FEE.—For all alike, $10.00. The admission fee is paid but once, and entitles the student the privileges of permanent membership in any department of the University. The annual tax is paid the first year, aud every year thereafter while at the University.

For further particulars, address the Dean of the Dental College, Ann Arbor, Michigan.

J. TAFT, Dean.

May-6t

### OHIO COLLEGE of DENTAL SURGERY

### 29 College Street.

### Thirty-Fourth Annual Session 1879-80

---

### FACULTY.

| | |
|---|---|
| A J S. TAYLOR, M. D., D. D. S., | Principles and Practice and Dental Hygiene. |
| WM. CLENDENIN, M. D., | Descriptive Anatomy. |
| J. S. CASSIDY, M. D., D. D. S., | Chemistry and Materia Medica. |
| J. L. CILLEY, M. D., | Physiology and Histology. |
| H. A. SMITH, D. D. S., | Clinical Dentistry. |
| A. O. RAWLS, D. D. S., | Pathology and Therapeutics. |
| J. R. CLAYTON, D. D. S., | Mechanical Dentistry and Metallurgy. |
| WM. TAFT, M. D., D. D. S., | Oral Surgery and Practical Anatomy. |
| OTTO ARNOLD, D. D. S., | Demonstrator of the use of Nitrous Oxide Gas. |

### DEMONSTRATORS.

| | |
|---|---|
| C. I. KEELY, | Demonstrator of Operative Dentistry. |
| H. L. MOORE, D. D. S., | Assistant Demonstrator of Operative Dentistry. |
| E. W. ANDERSON, D. D. S., | Demonstrator of Mechanical Dentistry. |
| C. W. WARDLE, D. D. S., | Instructor in Continuous Gum Work. |
| OSCAR HEISE, D. D. S., | Assistant to Chair of Chemistry. |

### CLINICAL INSTRUCTORS.

Dr. F. H. REHWINKEL,

Dr. G. W. KEELY,

Dr. J. I. TAYLOR,

Dr. F. PEABODY,

Dr. E. OSMOND,

Dr. N. S. HOFF,

Dr. J. E. CRAVENS,

Dr. A. W. SMITH,

Dr. F. A. HUNTER,

Dr. S. WARDELL,

Dr. C. R. TAFT,

Dr. D. W. CLANCEY.

Dr. W. S. HOW.

Dr. T. S HACKER.

This institution being *strictly a Dental College*, the lectures in all the branches taught, Anatomy, Physiology, Pathology, Therapeutics and Materia Medica included, are arranged with the view to qualify the student to intelligently pactrice his profession.

The College Building was designed, erected and is *exclusively used* for a Dental School. The location is central and within a short distance of the Post Office, Music and Exposition Hall, Hospital and other public structures. Located in one of the most populous cities, where there is no other Dental College, the supply of patients for clinics is abundant. Superior advantages are thus afforded students for practical work.

### REQUIREMENTS FOR GRADUATION.

A candidate for graduation must be twenty-one years of age. He must have attended two courses of lectures in a Dental or Medical College, the last of which must be in this institution. A reputable dentist in actual practice five years, inclusive of the term of pupilage, may be examined for the degree of D. D. S., after attending one full course of lectures.

### FEES.

| | |
|---|---|
| Matriculation Fee (but once) | $ 5 00 |
| Professor's Tickets for one session, | 75 00 |
| Demonstrator's Ticket (for Anatomy) | 5 00 |
| Diploma Fee, | 20 00 |

The session begins the 22d of October, and continues until the first of March. The regular lectures begins at once, and students should be prompt in matriculating.

Good Board can be had for $3 50 to $6 00 per week.

For further information, address

***H. A. SMITH, Dean,***
286 Race Street, Cincinnati.

### THE DENTAL COLLEGE
### OF THE
### UNIVERSITY OF MICHIGAN.

The Fifth Annual Session of this Institution will commence on the 1st of October and close on the last Wednesday of March, thus making a course of six months. The regular course of instruction commences at once, and will proceed through the term, with the customary vacation.

The students in this department will receive instructions in Anatomy, Physiology Pathology, Chemistry, Materia Medica, Therapeutics and Surgery, from the Professors of their respective branches in the *Department of Medicine and Surgery* of the University, when lectures commence, and continue the same as with the Dental College. There will also, in addition, be a special course upon each of these branches. These courses will each embrace from fifteen to twenty lectures.

---

### *FACULTY OF THE DENTAL DEPARTMENT.*

J. B. ANGELL, LL. D., - - - - - - - - President
J. TAFT, D. D. S., - Principles and Practice of Operative Dentistry
CORYDON L. FORD, M. D., D, D. S. - Anatomy and Physiology
J. A. WATLING, D. D. S., - - Clinical and Mechanical Dentistry
W. H. DORRANCE, D. D. S., Demonstrator of Mechanical Dentistry

### *SPECIAL COURSES.*

A. B. Palmer, M. D., - - - - - - Dental Pathology
Donald Maclean, M. D., - - - - - - Oral Surgery
E. S. Dunster, M. D.,
Diseases of Women and Children with reference to the Teeth
Geo. E. Frothingham, M. D. - - - - Dental Therapeutics

*Geo. L. Field, D. D. S., will give Special Instruction in Continuous Gum Work.*

Students should be promptly present at the College on Tuesday, September 30th, at ten o'clock, A. M., to make the preliminary arrangements for entering upon regular work on the morning of October 1st. Seats in the lecture room are assigned by selection to students in the order of registration on the Steward's books; and each student is expected to occupy during the session such seat as he may select. Students on arriving in Ann Arbor, should call at the Steward's office.

### *CONDITIONS OF GRADUATION.*

The candidate must be twenty-one years of age. He must furnish evidence of good moral character.

He must devote three years to the study of his profession. He must attend two full courses of lectures in the Dental College, or one course elsewhere and the last one here, and we recommend that he attend three courses regularly.

He must sustain an examination satisfactory to the Faculty in all the branches taught.

A graduate of the Medical College may enter the senior class, and if found qualified, may graduate after one year has been devoted to the study of dentistry.

### FEES AND EXPENSES.

The fees, which must be paid in advance, are as follows:

Residents of Michigan.—Matriculation fee, $10.00; annual dues, $20.00.

Non-Residents.—Matriculation, $25.00; annual dues, $25.00.

Graduation Fee.—For all alike, $10.00. The admission fee is paid but once, and entitles the student the privileges of permanent membership in any department of the University. The annual tax is paid the first year, aud every year thereafter while at the University.

For further particulars, address the Dean of the Dental College, Ann Arbor, Michigan.

J. TAFT, Dean.

May-6t

### OHIO COLLEGE of DENTAL SURGERY

### 29 College Street.

### Thirty-Fourth Annual Session 1879-80

---

### FACULTY.

Jas. TAYLOR, M. D., D. D. S., - Principles and Practice and Dental Hygiene.
WM. CLENDENIN, M. D., - - - - - - Descriptive Anatomy.
J. S. CASSIDY, M. D., D. D. S., - - - - Chemistry and Materia Medica.
J. L. CILLEY, M. D., - - - - - - - Physiology and Histology.
H. A. SMITH, D. D. S., - - - - - - - - Clinical Dentistry.
A. O. RAWLS, D. D. S., - - - - - - Pathology and Therapeutics.
J. R. CLAYTON, D. D. S., - - Mechanical Dentistry and Metallurgy.
WM. TAFT, M. D., D. D. S, - - Oral Surgery and Practical Anatomy.

### DEMONSTRATORS.

C. I. KEELY, D. D. S., - - - - - Demonstrator of Operative Dentistry.
OTTO ARNOLD, D. D. S., - Demonstrator of the use of Nitrous Oxide Gas.
H. L. MOORE, D. D. S., - Assistant Demonstrator of Operative Dentistry.
E. W. ANDERSON, D. D. S., - - Demonstrator of Mechanical Dentistry
C. W. WARDLE, D. D. S., - - - - Instructor in Continuous Gum Work
OSCAR HEISE, D. D. S., - - - - Assistant to Chair of Chemistry.
A. G. ROSE, D. D. S., - - - - - - Assistant to Chair of Physiology.

### CLINICAL INSTRUCTORS.

Dr. F. H. REHWINKEL, Dr. A. W. SMITH,
Dr. G. W. KEELY, Dr. F. A. HUNTER,
Dr. J. I. TAYLOR, Dr. S. WARDELL,
Dr. F. PEABODY, Dr. C. R. TAFT,
Dr. E. OSMOND, Dr. D. W. CLANCEY.
Dr. N. S. HOFF, Dr. W. S. HOW.

This institution being *strictly a Dental College*, the lectures in all the branches taught, Anatomy, Physiology, Pathology, Therapeutics and Materia Medica included, are arranged with the view to qualify the student to intelligently pactrice his profession.

The College Building was designed, erected and is *exclusively used* for a Dental School. The location is central and within a short distance of the Post Office, Music and Exposition Hall, Hospital and other public structures. Located in one of the most populous cities, where there is no other Dental College, the supply of patients for clinics is abundant. Superior advantages are thus afforded students for practical work.

### REQUIREMENTS FOR GRADUATION.

A candidate for graduation must be twenty-one years of age. He must have attended two courses of lectures in a Dental or Medical College, the last of which must be in this institution. A reputable dentist in actual practice five years, inclusive of the term of pupilage, may be examined for the degree of D. D. S., after attending one full course of lectures.

### FEES.

| | |
|---|---|
| Matriculation Fee (but once) | $ 5 00 |
| Professor's Tickets for one session, | 75 00 |
| Demonstrator's Ticket (for Anatomy) | 5 00 |
| Diploma Fee, | 20 00 |

The session begins the 22d of October, and continues until the first of March. The regular lectures begins at once, and students should be prompt in matriculating.

Good Board can be had for $3 50 to $6 00 per week.

For further information, address

***H. A. SMITH, Dean,***

286 Race Street, Cincinnati

### CONTENTS.

COMMUNICATIONS.

The New and the Old and the New Departure by an Old One. *By J. A. Robinson, D. D. S.* ........ 493

Historical Sketch of the Discovery of the Composition of Water. *By E. G. Betty, D. D. S.* ........ 506

Dental Students. *By D. C. Hawxhurst* ........ 514

Dr. Crawford W. Long, the Discoverer of Anæsthesia. Anæsthesia and Anæsthetics ........ 518

Trophic Neuralgias. *By John J. Caldwell, Baltimore, Md* ........ 521

SELECTIONS.

How Far Can We Hear with the Telephone? ........ 522

Air as a Stimulant ........ 523

India Rubber ........ 524

Statistics of Age ........ 524

Scientific News ........ 525

EDITORIAL.

The Gardner Case—Correction ........ 528
Bibliographical ........ 532

### *THE DENTAL REGISTER,*

A MONTHLY MAGAZINE DEVOTED TO THE INTERESTS OF

THE DENTAL PROFESSION.

*Terms Reduced to $2 50 per Year. Single Copies 25c.*

*EDITED BY PROF. J. TAFT, D. D. S.,*

AND PUBLISHED BY

SPENCER & CROCKER, Ohio Dental Depot.

The volume commences in January and closes in December.

The REGISTER being issued monthly is always fresh, therefore Indispensable to the practitioner who desires to keep posted up with the new inventions and improvements which are daily developed. Neither expense nor effort will be spared to give value and variety to its contents. Articles will be illustrated with well executed engravings whenever they will add to the interest or assist to explanation.

Its extensive circulation and frequency of issue make it one of the BEST DENTAL ADVERTISING MEDIUMS in the United States.

All subscrptions must begin with January or July.

Local or special notices, five lines of less, will be inserted for $3.00 each insertion; over five lines, 50 cts. per line.

All advertising bills payable in advance, or at furthest, after the issue of the first number containing the advertisement. All transient advertisements to be paid for in advance.

☞CONTRIBUTIONS SOLICITED.

Exclusively Editorial Communications should be addressed to Editor.

The latest number of the REGISTER may be ordered with or without other goods at any time, and all letters should be addressed to

**SPENCER & CROCKER, Ohio Dental Depot, Cincinnati, O.**

